# Successful carotid endarterectomy in a patient with an aberrant branch from the common carotid artery

**DOI:** 10.1308/003588413X13511609955139

**Published:** 2013-03

**Authors:** YC Chan, WH Wong, SW Cheng

**Affiliations:** Queen Mary Hospital,Hong Kong

**Keywords:** Common carotid anomaly, Asymptomatic stenosis, Endarterectomy, Superior thyroid artery

## Abstract

We report a patient who had an 80% asymptomatic stenosis in the distal right common carotid artery with an incidental finding of an aberrant branch arising from the right common carotid artery. He underwent an elective right carotid endarterectomy with an uneventful recovery. This is the first case in the literature of a successful endarterectomy in a patient with a common carotid anomaly and it emphasises the importance of careful dissection for unexpected anatomy.

The common carotid artery does not typically have any branches in the neck before it bifurcates into the external and internal carotid arteries. Anomaly of the common carotid artery is rare and is usually an incidental finding in postmortem anatomical dissection.^1^ These anomalies may be associated with high bifurcation of the common carotid arteries at the level between the second and the third cervical vertebrae, giving rise to aberrant branches below the bifurcation.^2^

Reported cases in the literature are few, and the aberrant branch from the common carotid artery may represent the superior thyroid artery,^3,4^ the superior thyroid artery and the ascending pharyngeal artery from a common trunk,^5^ the ascending pharangeal branches,^2^ the thyrolingual trunk^6^ or the inferior thyroid artery.^7^ We report the case of a 73-year-old man who presented with an 80% asymptomatic right carotid stenosis. He was found to have an aberrant branch from the common carotid artery. To our knowledge, this is the first report in the literature of a patient who had an aberrant branch from the common carotid artery who underwent a successful carotid endarterectomy.

## Case history

**Figure 1:**
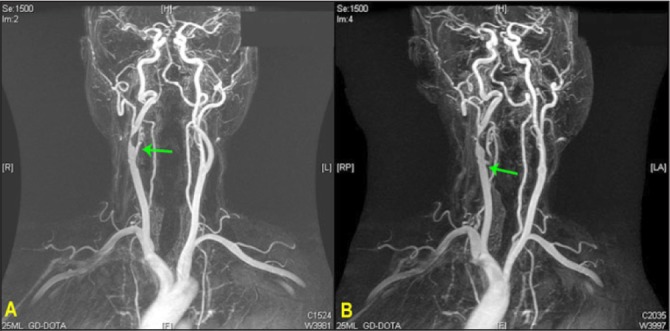
Preoperative magnetic resonance angiography showing an 80% stenosis in the distal right common carotid artery with an incidental finding of an aberrant branch of the right common carotid artery. The anteroposterior view is represented (A) as well as the 30° right posterior view (B), showing clearly the origin of the superior thyroid artery from the right common carotid artery.

A 73-year-old man presented with a few weeks’ history of neck discomfort and stiffness. Cervical spinal radiography showed degenerative changes with narrowing of the C5 and C6 disc space. Physical examination showed a right carotid bruit and magnetic resonance angiography revealed an 80% stenosis in the distal right common carotid artery. There was an incidental finding of an aberrant branch of right common carotid artery (Fig 1).

**Figure 2:**
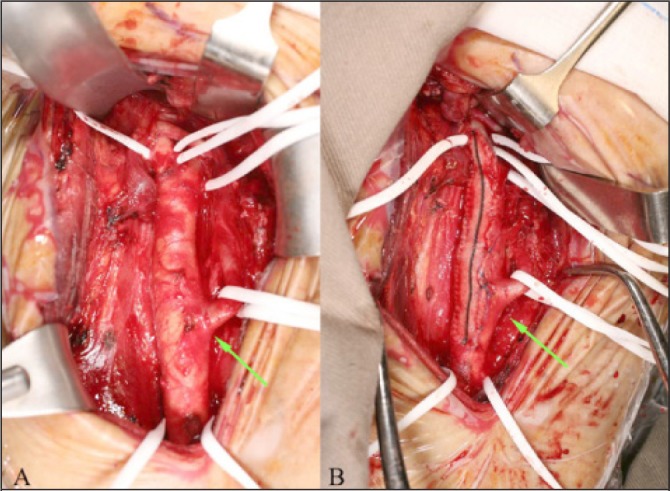
Intraoperative photographs showing the aberrant superior thyroid branch prior to endarterectomy (A) and that this branch was preserved intact after completion of the endarterectomy and with dacron patch closure of the arteriotomy (B)

**Figure 3:**
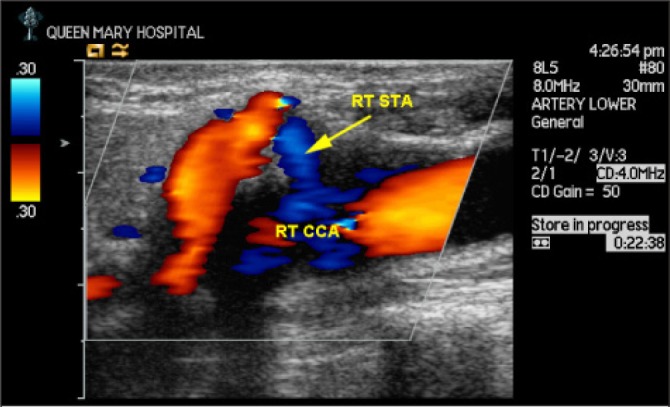
Postoperative duplex ultrasonography showing that there was no restenosis in the common and internal carotid arteries, and that the aberrant branch (arrowed) remained widely patent with a velocity of 88cm/s RT STA = right superior thyroid artery; RT CCA = right common carotid artery

The patient underwent an elective right carotid endarterectomy under regional anaesthesia and the aberrant branch was identified to be the superior thyroid artery, associated with a high bifurcation of the common carotid artery above the angle of the mandible (Fig 2a). A shunt was not required during the operation and the arteriotomy was closed with a dacron patch (Fig 2b). The operation was uneventful and the patient remained well in the postoperative period. Postoperative duplex ultrasonography surveillance showed that there was no restenosis in the common and internal carotid arteries, and that the aberrant branch remained widely patent with a velocity of 88cm per second (Fig 3). The high common carotid bifurcation into the internal and external carotid arteries could also be identified clearly.

## Discussion

This is the first report in the literature of an incidental finding of an aberrant superior thyroid artery arising from the right common carotid artery in a patient who had a successful carotid endarterectomy. It demonstrates that surgeons involved in head and neck surgery should be aware of anatomical variations as the common carotid artery does not usually have any branches in the neck before it bifurcates into the external and internal carotid arteries. This patient in particular had a high common carotid bifurcation.

The point at which the common carotid artery bifurcates is highly variable.^8^ In a series of 76 carotid dissections in human cadavers, Lo *et al* found that when the common carotid artery had a relatively low bifurcation, such as at the lamina of thyroid cartilage, the superior thyroid artery tended to originate from the external carotid artery.^9^ In contrast, when the common carotid artery had a high bifurcation, the superior thyroid artery tended to originate at the level of the carotid bifurcation. In this series, none of the common carotid arteries had aberrant branches. With the complex variation of common carotid artery bifurcation, the origin of the superior thyroid artery could be highly variable.

A review of the literature showed that the superior thyroid artery arising from the common carotid artery is in fact not as rare as one may think. Kukwa and Zbrodowski were the first to report a case of origin of the superior thyroid artery from the left common carotid artery in 1966.^10^ Fujimoto *et al* reported a case of the left superior thyroid artery arising from the left common carotid artery in 1974.^3^ In 1978 Smith and Benton reported an anomalous right superior thyroid artery arising from the common carotid artery 27mm proximal to the bifurcation.^11^ Akyol *et al* reported a superior thyroid artery arising from the common carotid artery in 1997.^4^

Historically, anatomical textbooks and dissertations have long quoted a much higher incidence of superior thyroid artery arising from the common carotid artery. Quain showed in 1844 that 41 out of 292 (14%) cadaveric dissections had the superior thyroid artery arising from the common carotid artery.^12^ In 1903 Livini showed that in 18 out of 200 cases (9%) the superior thyroid artery arose from the common carotid artery.^13^ Similar cases were reported by Poynter in 1922 (14 cases in 200 [7%]),^14^ by Adachi in 1928 (39 cases in 300 [13%]),^15^ by Aaron and Chawaf in 1967 (24 cases in 187 [13%]),^16^ by Poissel and Golth in 1974 (10 cases in 156 [6%])^17^ and by Lucev *et al* in 2000 (19 cases in 40 [47%]).^18^

In a 4-year study of dissecting 330 heminecks in 165 human cadavers, Vázquez *et al* also demonstrated that there were many different variations in origin of the superior thyroid artery.^19^ In this study, the superior thyroid artery was found to arise from the common carotid artery in 55 cases (27%), being more common on the left side. In these cases, the origin of the superior thyroid artery from the common carotid artery was usually situated at a distance of 0.1–2.1cm from the carotid bifurcation. In a study of 49 cadaveric carotid triangles, Hayashi *et al* showed that 30% of the superior thyroid arteries arose from the distal common carotid artery.^20^

In all of the above cases or case series, the origin of the superior thyroid artery appeared to be related to the level of common carotid bifurcation. When the carotid artery had a relatively low bifurcation, the superior thyroid artery tended to originate from the external carotid artery.^21^ In contrast, when the common carotid artery had a high bifurcation, the superior thyroid artery tended to originate at the level of carotid bifurcation or from the common carotid artery.

## Conclusions

To our knowledge, this is the first reported case in the literature of a patient who underwent a successful carotid endarterectomy with an aberrant superior thyroid artery from the common carotid artery. Although anatomical textbooks and cadaveric dissections showed that this anomaly may not be as rare as anticipated, our case corresponds well with anatomical finding in the literature. This patient had a high right carotid bifurcation above the level of angle of the mandible and the origin of the superior thyroid artery originated from the mid-common carotid artery. For surgeons performing the carotid endarterectomy, the aberrant branch may be mistaken for the external carotid artery. In our patient, the location of the plaque appeared quite low and this may conceivably be due to turbulence at this large branch. This case highlights that awareness of anatomical variations is important to prevent morbidity and mortality in carotid artery operations.
